# Expert consensus to optimize the management of older adult patients with advanced EGFR-mutated non-small cell lung cancer

**DOI:** 10.1007/s12094-023-03286-3

**Published:** 2023-08-11

**Authors:** Ernest Nadal, Martín Oré-Arce, Jordi Remon, Reyes Bernabé-Caro, Marta Covela-Rúa, Javier de Castro-Carpeño, Bartomeu Massutí-Sureda, Mónica Guillot-Morales, Margarita Majem, Inmaculada Maestu-Maiques, Idoia Morilla-Ruíz, Regina Gironés

**Affiliations:** 1 Department of Medical Oncology, Institut Català d’Oncologia (ICO), Institut d’Investigacions Biomèdiques de Bellvitge (IDIBELL), Duran i Reynals University Hospital, Barcelona, Spain; 2 Department of Medical Oncology, Marina Baixa de Villajoyosa Hospital, Alicante, Spain; 3 Department of Medical Oncology, HM Nou Delfos Hospital, Barcelona, Spain; 4 Department of Medical Oncology, Virgen del Rocío University Hospital, Sevilla University, Seville, Spain; 5 Department of Medical Oncology, Lucus Augusti University Hospital, Lugo, Spain; 6 Department of Medical Oncology, La Paz University Hospital, IdiPAZ, Madrid, Spain; 7 Department of Medical Oncology, Alicante General University Hospital, Alicante, Spain; 8 Department of Medical Oncology, Son Espases University Hospital, Palma, Spain; 9 Department of Medical Oncology, Santa Creu i Sant Pau Hospital, Barcelona, Spain; 10 Department of Medical Oncology, Dr. Peset University Hospital, Valencia, Spain; 11 Department of Medical Oncology, Navarra University Hospital-NavarraBioMed, IdisNa, Pamplona, Spain; 12 Department of Medical Oncology, Polytechnic la Fe University Hospital, Avinguda de Fernando Abril Martorell, 106, 46026 Valencia, Valencia Spain

**Keywords:** Lung cancer, *EGFR* mutation, Older adult patient, Targeted therapy, TKI, Geriatric assessment

## Abstract

Lung cancer (LC) is associated with ageing, with the average age of affected individuals being approximately 70 years. However, despite a higher incidence and prevalence among older people, the older adult population is underrepresented in clinical trials. For LC with Epidermal Growth Factor Receptor (*EGFR*) mutations, there is no clear association of this mutation with age. Geriatric assessments (GAs) and a multidisciplinary approach are essential for defining the optimal treatment. In this consensus, a group of experts selected from the Oncogeriatrics Section of the Spanish Society of Medical Oncology (Sección de Oncogeriatría de la Sociedad Española de Oncología Médica—SEOM), the Spanish Lung Cancer Group (Grupo Español de Cáncer de Pulmón—GECP) and the Association for Research on Lung Cancer in Women (Asociación para la Investigación del Cáncer de Pulmón en Mujeres—ICAPEM) evaluate the scientific evidence currently available and propose a series of recommendations to optimize the management of older adult patients with advanced LC with *EGFR* mutations.

## Introduction

Scientific advances, progress in disease treatments and improvements in health conditions led to a progressive increase in the life expectancy worldwide. According to data from “World Population Prospects 2019” released by the United Nations, by the year 2050, one in every six people in the world will be over 65 years old (17%), much higher than that for the year 2019 (9%, one in every 11 people) [1].

Ageing brings with it an increased risk of cancer, originating from the accumulation ofoxidative stress and DNA damage across the years, both by endogenous and exogenous factors. The accumulation of free radicals, exposure to smoking, environmental pollution, changes in diet, etc., all increase the risk of cancer [2]. Also, with age, senescent cells accumulate, which can alter the microenvironment and promote tumour development. In addition, in older adults, there is a progressive deficit in immune function, altering the immune response to tumour growth [3]. Currently, developed countries considered 70 years old as the cut-off age for older adult patients. However, the majority of subanalysis had used 65 years old as a cut-off age.

Lung cancer (LC) is one of the neoplasms most associated with ageing. In this pathology, the risks described above accumulate.

The median age of patients with cancer is close to 70 years, but 30% are elder at diagnosis (almost 10% of patients are older than 80 years at diagnosis) [4]. Despite the higher incidence and prevalence of cancer among older individuals, the older adult population is often underrepresented in LC clinical trials. They are usually excluded due to comorbidity criteria or even due to the bias or ageism of professionals, who are less likely to include older adult patients in clinical trials.

The development of new drugs, especially targeted therapies, has made possible to change the prognostic and therapeutic paradigm of the cancer population. These advances must be employed throughout the population, regardless of chronological age [5]. For this reason, a group of experts from the Oncogeriatrics Section of the Spanish Society of Medical Oncology (Sección de Oncogeriatría de la Sociedad Española de Oncología Médica—SEOM), the Spanish Lung Cancer Group (Grupo Español de Cáncer de Pulmón—GECP) and the Association for Research on Lung Cancer in Women (Asociación para la Investigación del Cáncer de Pulmón en Mujeres—ICAPEM) reviewed the scientific evidence available for the older adult population with advanced LC, specifically in the population subgroup with activating mutations in the epidermal growth factor receptor (EGFR).

## Epidemiological characteristics of older adult patients with *EGFR*-mutated LC

Although some studies indicate a higher incidence of *EGFR*-mutated LC among older adult patients [6], current data do not support a significant relationship between presence of mutation and ageing. Indeed, a recently published meta-analysis ruled out a relationship between age and a higher probability of *EGFR* gene mutation [7]. However, this mutation is associated with gender and smoking. Thus, while for men the incidence of mutated *EGFR* remains similar with age, for women, the incidence of mutated *EGFR* increases after the age of 65 [8]. Among never-smoker patients, *EGFR* mutations tend to increase with age in both sexes. The deletion of exon 19 is more frequent in patients under 50 years, while the L858R mutation in exon 21 is more common after 61 years [9]. There is no differential clinical profile of *EGFR*-mutated LC in the older adult population [10].

## Evidence from geriatric assessments in the older adult population with LC

### Geriatric assessments of cancer patients

The management of older adult patients with cancer is challenging due to the heterogeneity associated with ageing because chronological age does not always correspond to biological age. Over the years, there is a decrease in the functional reserve, leading to an increased risk of complications in situations of stress or illness. Additionally, the frequent presence of comorbidities, polypharmacy and malnutrition in this population can affect treatment tolerance and impact disease evolution (Table 1).

**Table 1:** Aspects to consider in GA

Medical evaluation	Comorbidities, polypharmacy, nutritional status, geriatric syndromes
Functional evaluation	ADL, IADL
Psychological evaluation	Cognitive function, psychological state
Social evaluation	Socioeconomic status

*ADL* activities of daily living, *GA* geriatric assessment, *IADL* instrumental activities of daily living

Since 2005, the International Society of Geriatric Oncology (SIOG) has recommended a geriatric assessment (GA) for older adult patients with cancer [11]. This recommendation also appears in the main clinical treatment guidelines of the National Comprehensive Cancer Network® (NCCN) [12], the European Society for Medical Oncology (ESMO) [13], and the SEOM [14, 15]. An adequate GA must cover functional, cognitive, and emotional aspects, nutritional status, comorbidities, social situation, and the presence of geriatric syndromes [11, 15]. GA allows the detection of problems and identification of needs in different spheres, the development of a treatment plan, an estimation of survival, and predictions of toxicity, while taking into account the preferences of the patient [16].

GA has been shown to provide more information than functional scales, such as the Eastern Cooperative Oncology Group (ECOG) performance status (PS) or the Karnofsky index, which are frequently used in daily clinical practice [17]. Given that the population of older patients with cancer is increasing and that GA requires considerable time to perform, a series of screening tools have been developed to select patients who require a more complete and specific assessment. The most widely used tool in oncology is the G8 scale, which was developed specifically for use in older patients with cancer [18].

Once a GA is completed, patients are classified as robust, prefragile and fragile [19]. GA, in addition to allowing treatment to be adapted to the profile of each patient, allows for the identification of dose adjustments or reductions and alternative treatment options, for example, granulocyte colony-stimulating factors (G-CSF), especially for individuals indicated for chemotherapy and at a high risk of toxicity [14].

### GA in patients with LC, including EGFR-mutations

**Fig. 1:**
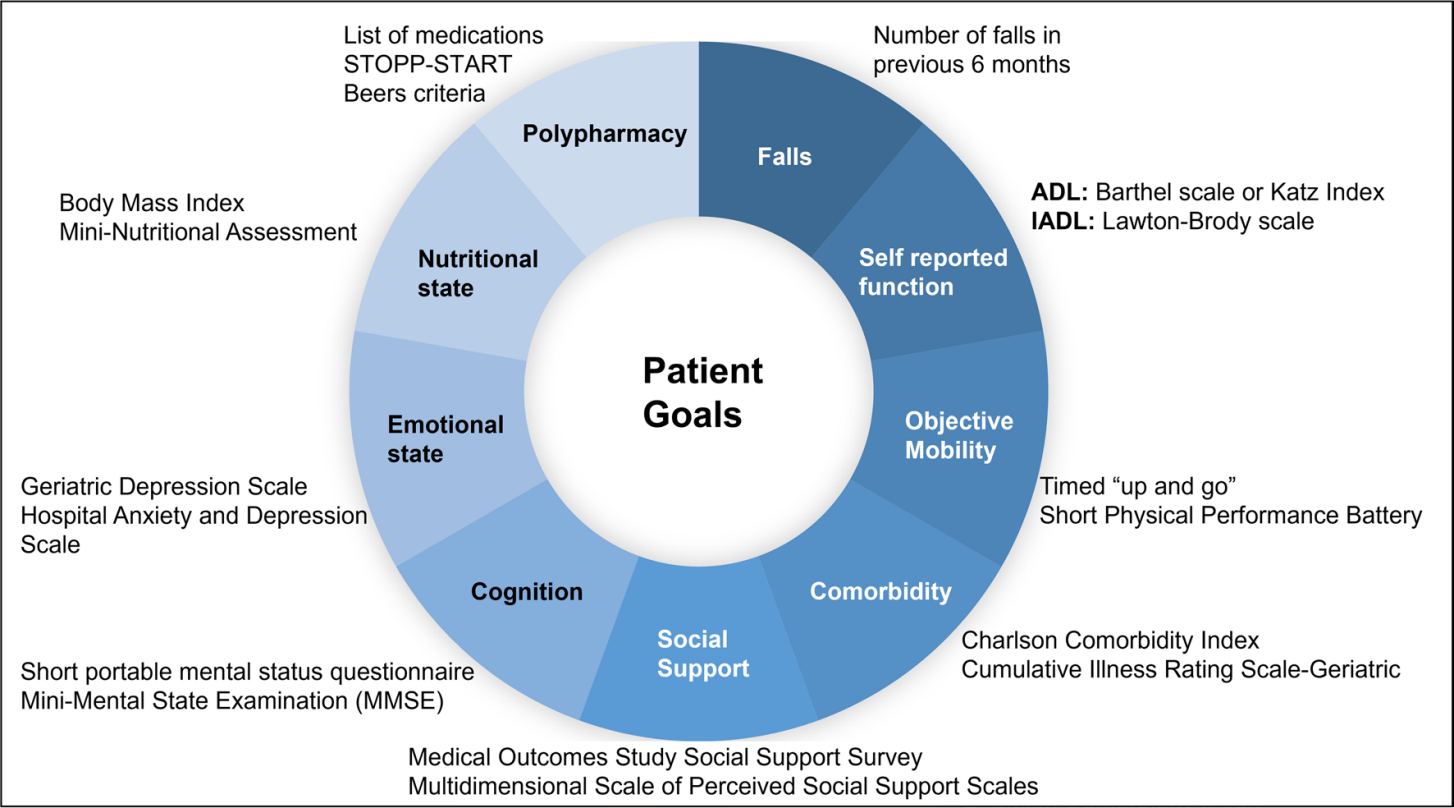
Domains and tools for the GA of the patient with LC. *ADL* activities of daily living, *GA* geriatric assessment, *IADL* instrumental activities of daily living, *LC* lung cancer, *MMSE* Mini-Mental State Examination

Evidence supports the use of GA to evaluate vulnerabilities in older patients with LC. Instrumental activities of daily living (IADL) identify one of the most frequently detected impairments, followed by polypharmacy, comorbidity, risk of malnutrition and depression. GA classified 37% of evaluated patients with PS 0, and 33% with PS 1, as vulnerable because of comorbidity; and 11% and 19%, respectively, as frail because of activities of daily living (ADL) and comorbidity, respectively (Fig. 1) [20].

A systematic review demonstrated the association between GA and outcome in patients treated with chemotherapy not assessed for PS [21]. Patients with a median age of 76 years had a high prevalence of geriatric impairments (29% cognitive, 70% IADL). Objective physical capacity and nutritional status had a consistent association with mortality and with chemotherapy completion.

A phase III trial randomized 494 patients ≥ 70 years, with PS 0–2 and stage IV non-small cell lung cancer (NSCLC) [22], to a standard strategy of treatment allocation based on age and PS compared with an experimental arm based on GA. All patients were *EGFR* and *ALK* wild type or unknown. In the control arm, patients received double or single chemotherapy agent according to PS and age. In the experimental arm, fit and vulnerable patients received double or single agent, respectively, and frail patients received best support care. Patients in the GA arm experienced significantly less all-grade toxicities (86% vs. 93%, respectively; *p* = 0.015). However, no differences in terms of overall survival (OS) were observed.

In older adult patients with LC harbouring an *EGFR* mutation, several observational studies support the safety and efficacy of EGFR tyrosine kinase inhibitors (TKIs) in first line, but there are concerns about a higher risk of toxicity. Frailty has been retrospectively evaluated in 114 patients ≥ 80 years treated with EGFR TKIs. GA was performed in 35% of them. Twenty-eight percent had PS 3–4, 45% needed assistance at home and 38% were taking ≥ 6 drugs per day. There was a clear benefit of EGFR TKIs in terms of survival, and toxicities were consistent with adverse events (AEs) observed in younger patients [23]. A recent systematic review recommends GA to identify malnutrition, comorbidities, polypharmacy, and an alteration of the functional status, which might impact OS and progression free survival (PFS) [24].

### Polypharmacy due to comorbidities and drug interactions

The existence of drug interactions is relevant when initiating treatment with EGFR TKIs in older adult patients with LC, as this population is prone to polypharmacy due to the symptoms of cancer and the presence of comorbidities. In a retrospective study of 334 patients treated with EGFR TKIs, a prevalence of polypharmacy of 38% was observed, and potentially inappropriate medication (PIM) was detected for 32% of the participants [25]. Polypharmacy was independently correlated with OS and hospitalization during treatment.

Most EGFR TKIs are susceptible to drug interactions throughout the pharmacokinetic process. The alteration in gastric pH affects the absorption of erlotinib, gefitinib and dacomitinib. Among older adult patients with cancer, approximately 55% use proton pump inhibitors [26], which have been associated with a lower plasma concentration and OS in patients treated with erlotinib [27, 28].

EGFR TKIs are substrates of membrane transporters, such as *P-*gp and breast *cancer resistance protein* (BCRP). The inhibition or induction of these membrane transporters by different drugs can influence the absorption of EGFR TKIs. EGFR TKIs can increase exposure to BCRP substrates, such as rosuvastatin, and P-gp substrates, such as digoxin, dabigatran, aliskiren and pravastatin, which have a narrow therapeutic index [29].

Although the metabolism of some EGFR TKIs begins in the intestine, most are primarily metabolized in the liver. The enzyme most involved is cytochrome CYP3A4 [29]; its inhibition increases the pharmacokinetic parameters of erlotinib, gefitinib and osimertinib, and its induction decreases their plasma levels. Ciprofloxacin and strong CYP1A2 inhibitors can elevate erlotinib levels, which, together with gefitinib, can potentiate the effect of warfarin [30]. Although predictable drug interactions with direct anticoagulants, such as apixaban, may not be relevant [31], control of the therapeutic action is recommended because atrial fibrillation is the most frequent arrhythmia. Afatinib forms covalent adducts with proteins, and its enzymatic metabolism is minimal; therefore, it is not affected by cytochromes and should be considered in this context [29]. Finally, the risk of QTc interval lengthening observed with osimertinib is an important factor to consider in patients with cardiac risk factors and could increase when osimertinib is prescribed with other drugs, such as some antibiotics, antifungals, antidepressants and antiemetics [29].

Therefore, to avoid affecting efficacy and to reduce the risk of toxicity in older adult patients requiring EGFR TKIs, concomitant medication should be reviewed, and any PIM should be avoided. More studies are needed in this regard. Currently, the choice of EGFR TKI will depend on the alternatives that exist in each scenario and the concurrent medications essential for the treatment of each patient’s comorbidities.

## Treatment options for older adult patients with *EGFR*-mutated LC

Table 2 summarizes the efficacy and safety of different schedules to treat older adult patients with *EGFR*-mutated LC.

**Table 2:** Efficacy and toxicity of treatment for older adult patients with mutated *EGFR* LC

Study and phase	Patient characteristics	RR, PFS and OS	Main G3–4 toxicities
Gefitinib			
IPASS trial [34] Phase III	76 pts ≥ 65 yr	PFS: HR 0.58 95% CI 0.45–0.76; *p* < 0.001	NR
WJTOG3405 trial [35] Phase III	86 pts ≤ 75 yr	PFS: 9.2 vs*.* 6.3 m; HR = 0.489 No differences between > or < 65 yr	NR
NEJ 003 Study [36] Phase II	31 pts ≥ 75 yr Median age: 80 yr	RR: 74%; DCR: 90%; PFS: 12.3 m 2-yr S: 58.1%; 95% CI 45.2–70.9%	G3–4 AEs: 29%; G5 ILD: 1 pt G3–4 transaminases elevation: 19%
Phase II trial [37]	30 pts ≥ 80 yr or PS 3–4 Median age: 72 yr	RR: 66%; DCR: 90%; PFS: 6.5 m OS: 17.8 m; 1-yr S: 63% PS was improved in 68% of pts	G4 ILD: 1 pt G3 anaemia: 7% G3–4 transaminases elevation: 10%
Retrospective study [38]	62 pts ≥ 75 yr Median age: 80 yr	RR: 61%; DCR: 84%; PFS: 13.2 m OS: 19 m	G3–4: 29% pts Rash: 3%; Transaminases elevation: 21%
Erlotinib			
EURTAC [39] Phase III	173 pts 88 pts ≥ 65 yr	PFS: HR 0.37 PFS: HR: 0.28 in > 65 yr	NR
OPTIMAL [40] Phase III	82 pts 19 pts ≥ 65 yr	PFS: HR 0.16 PFS: HR 0.17 in > 65 yr	NR
ENSURE [41] Phase III	110 pts 45 pts ≥ 65 yr	No subanalysis for older adult pts	NR
Phase II trial [42]	32 pts ≥ 75 yr Median age: 80 yr	RR: 56%; DCR: 91% PFS: 15.5 m	G3–4: 28% pts G4 ILD: 1 patient
Phase II trial [43] Low dose or erlotinib	80 pts > 75 yr or frail Median age: 80 yr	RR: 60% DCR: 90% PFS: 9.3 m; OS: 26.2 m	G3–4: 18% pts Transaminases elevation: 5%
Phase III trial [44] Placebo vs erlotinib	770 pts; 63% > 75 yr Median age: 77 yr	PFS: HR 0.80; 95% CI 0.68–0.93; *p* = 0.0054; OS: No differences	G3–4 diarrhoea: 8% with erlotinib G3–4 rash: 23%
Afatinib			
LL3 subanalysis [47, 48]	LL3—134 pts ≥ 65 yr	PFS: 13.6 vs 8.2 m; HR 0.60 OS: 31.6 vs 24.9 m; HR 0.73; *p* = 0.22	G3-4 diarrhoea: 21% Rash/acne: 19%; Stomatitis: 10%
OS from LL3 and LL6 [48]	LL6—86 pts ≥ 65 yr	PFS: 13.1 vs 4.1 m; HR 0.17 OS: 23.2 vs 19.0 m; HR 0.60; *p* = 0.10	G3–4 diarrhoea: 8% Rash/acne: 9%; Stomatitis: 3%
Phase II [49] ≥ 70 yr, 30 mg dose	40 pts Median age: 77 yr	OR: 73%; DCR: 100%; PFS: 12.9 m 1-yr OS: 87%; 2-yr OS: 61% 3-yr OS: 52%	G3 pneumonitis 8%; 2 deaths for pneumonitis. Afatinib discontinued in 8 pts
Phase II [50] Afatinib 20 mg/day orally	53 pts Median age: 70 yr	RR: 66%; DCR: 93% PFS: 12.6 m	G ≥ 3: 23%, including diarrhoea in 4 pts
Dacomitinib			
ARCHER 1050 [51] Phase III	227 pts with dacomitinib 94 pts ≥ 65 yr	PFS ≥ 65: HR 0,69	No major toxicity
ARCHER 1009 [52] Phase III	227 pts with dacomitinib 65–74 yr (163); ≥ 70 yr (50)	PFS > 65 yr: 0.92; PFS > 75 yr: 0.96 No differences in pts > 65 yr	NR
RWD study [53]	56 pts 31 pts > 60 yr	PFS: 5.4 m OS: 14 m	NR
Osimertinib			
HOT2002 [58] Retrospective study	132 pts ≥ 75 yr	RR: 75%; DCR: 93% PFS: 19.4 m, better PFS in < 80 yr	G ≥ 3 higher than FLAURA trial (42% vs*.* 34%) Pneumonitis: 17%; G3 ≥ 3: 9% (13% mortality)
ASTRIS trial [59] T790M *EGFR* pre-treated	3014 pts 396 pts ≥ 75 yr	RR: 58% in ≥ 75 yr; PFS: 11.8 m 1-yr S: 74%	NR
Phase II trial [60] T790M *EGFR* pre-treated	36 pts ≥ 75 yr	RR: 58%; DCR: 97%; PFS: 11.9 m OS: 22.0 m	G ≥ 3 in 10 cases: 28% G3-4 pneumonitis: 6%
Phase II trial [61] T790M *EGFR* pre-treated	18 pts ≥ 75 yr	RR: 61%; PFS older adults: 17.7 m OS: 38.6 m; 95% CI 14.3–52.8; *p* = 0.20	G ≥ 2 paronychia (2% vs 17%; *p* = 0.04) Similar dose reduction and discontinuations
GFPC 01–2016 [62] T790M *EGFR* pre-treated	43 pts > 80 yr Median age: 85 yr	PFS: 17.5 m OS: 22.8 m	NR

*CI* confident interval, *CT* chemotherapy, *DCR* disease control rate, *EGFR* epidermal growth factor receptor, *G* grade, *HR* hazard ratio, *ILD* interstitial lung disease, *LL3* LUX-Lung 3, *LL6* LUX-Lung 6, *m* months, *NR* not reported, *OS* overall survival, *PFS* progression free survival, *PS* performance status, *pt* patient, *RR* response rate, *RWD* real world data, *S* survival, *yr* year

### First-generation drugs

Despite the rapid development of drugs targeting EGFR, there are few specific trials focused on their efficacy in older adult patients and most information comes from subgroup analyses in the major randomized clinical trials that supported the registration of the drugs.

#### Gefitinib

The first evidence of the efficacy of the targeted therapy gefitinib in LC *EGFR*-mutated patients were reported in 2004 [32, 33]. Results of phase III trials started in 2009 with the IPASS trial showing an increased PFS (9.5 vs. 6.3 months, hazard ratio [HR] 0.48) and a better response rate (RR) (71% vs. 47%) in patients with *EGFR*-mutated LC when treated with gefitinib compared with platinum-based chemotherapy [34]. Median age of included patients was 57 years and for 76 patients aged > 65 years results remained significant with HR 0.58 for PFS. A second phase III trial comparing gefitinib with carboplatin-docetaxel in 172 patients with a median age of 64 years achieved similar results (median PFS was 9.2 vs. 6.3 months, HR = 0.489) without any significant differences between patients older or younger than 65 years [35].

One phase II trial performed in 31 patients older than 75 years (with a median age of 80 years) showed a median PFS of 12.3 months with a RR of 74%. Disease control rate (DCR) was 90% with an incidence of grade 3–4 AEs of 29% [36]. Inferior results were seen in another phase II trial performed in 30 patients older than 80 years or with a PS 3–4 (median age: 72 years). PFS was only 6.5 months, OS was 17.8 months, RR was 66% and DCR was 90%. PS was improved in 68% of patients [37].

In a retrospective analysis of 62 patients older than 75 years with a median age of 80 years, results were PFS 13.2 months, OS 19 months, RR 61%, and DCR 84%. A total of 29% of patients showed grade 3–4 AEs [38].

#### Erlotinib

Phase III trials comparing erlotinib with platinum-based chemotherapy were: (i) the EURTAC trial with an HR of 0.37 in terms of PFS in favour of erlotinib in patients with a median age of 65 years. In 88 patients older than 65 years, HR was 0.28 [39]; (ii) the OPTIMAL trial with an HR of 0.16 in terms of PFS in patients with a median age of 58 years [40]. In the subgroup of patients older than 65 years, 38 of them (25%) had an HR of 0.17 when treated with erlotinib; and (iii) the ENSURE trial, which included only 45 patients (21%) aged more than 65 years, but it did not provide a specific subgroup for older adult patients [41].

Phase II trials with first-line erlotinib in patients older 75 years were: (i) Inoue et al*.* trial, 32 patients with a median age of 80 years who had a median PFS of 15.5 months, RR of 56%, DCR 91% with an incidence of grade 3–4 AEs observed in 28% of patients [42]; (ii) Miyamoto et al.trial, 80 patients (> 75 years or frail) with a median age 80 years (49–90) who achieved a median PFS of 9.3 months, an OS of 26.2 months, RR of 60%, DCR of 90%, and 18% of patients developing grade 3–4 AEs [43].

In a phase III trial that compared erlotinib with placebo in 770 unselected patients with advanced NSCLC unsuitable for chemotherapy (63% of them > 75 years), OS benefit was seen in patients who developed first-cycle rash in all subgroups including those with the worst characteristics, such as age ≥ 75 years [OS HR 0.77 (0.61–0.97, *p* = 0.028) and PFS HR 0.71 (0.56–0.89, *p* = 0.0032)] [44].

In a pharmacokinetics study of erlotinib plasma concentrations, age was associated with a lower oral clearance (CL/F), and a dose reduction or treatment discontinuation due to AEs was observed in 52% of patients > 75 years old compared with 25% of younger patients [45].

A meta-analysis, assessing the impact of different *EGFR* mutations and clinical characteristics on PFS in 1649 patients with advanced *EGFR*-mutant NSCLC included in randomized controlled trials of *EGFR* TKIs compared with chemotherapy in first-line treatment (203 of them treated with gefitinib and 278 with erlotinib in addition to 472 treated with afatinib), found that age (lower or greater than 65 years) did not predict a significant difference in terms of PFS, with HR 0.34 in younger versus 0.37 for overall population with interaction p-value of 0.27 [46].

### Second-generation drugs

Currently, the second-generation TKIs available for the treatment of patients with *EGFR*-mutated LC are afatinib and dacomitinib.

#### Afatinib

In the Lux LUNG 3 and Lux LUNG 6 phase III studies of afatinib, 134 patients and 86 patients older than 65 years were included, and RR, PFS and OS results for these patients were similar to those observed in the general population, with a similar percentage of Grade 3 AEs, the most frequent being gastrointestinal (GI) toxicity, specifically diarrhoea, and dermal and nail toxicity [47, 48].

The toxicity observed in the phase III studies was the basis for the design of phase II studies with a population ≥ 70 years. A prospective study by Imai et al*.* included 40 patients with a median age of 77 years [49] who were initially administered 30 mg/day of afatinib. A RR of 73% was observed, with a median PFS of 12.9 months and percentages of patients alive in the first, second and fourth years of 87%, 61% and 52%, respectively. The most important grade 3 toxicities were diarrhoea (13%) and mucositis (8%). Two toxic deaths from pneumonitis were reported, and eight patients had to discontinue treatment.

A second study evaluated the administration of a starting dose of 20 mg/day of afatinib in patients with a mean age of 70 years. In that study, a RR of 66% was observed, with a PFS of 12.6 months, like previous studies with higher doses of afatinib. The incidence of GI toxicity (grade 3 diarrhoea) was 7.5%, which forms the basis of starting treatment with afatinib at low doses to maintain efficacy while reducing GI toxicity [50].

#### Dacomitinib

A phase III study comparing dacomitinib with gefitinib (ARCHER 1050) included 94 patients (41%) in the dacomitinib arm, without greater toxicity and with similar efficacy to the other treatment arm [51]. In a phase III study comparing dacomitinib with erlotinib (ARCHER 1009), 163 pretreated patients aged 65–74 years (37%) and 50 patients older than 70 years (11%) were included [52]. No differences were observed in terms of efficacy between the population older than 65 years and the overall study population.

For dacomitinib, there are no clinical trials with a population ≥ 70 years, but there are studies with real world data (RWD) in which dacomitinib has been administered to patients up to 83 years of age. In these studies, the starting dose was adjusted to 30 mg/day in patients with PS 1 and with a starting weight less than 60 kg, but no dose adjustment was made only according to patients’ age [53].

### Third-generation drugs

#### Osimertinib

No data in patients ≥ 70 years have been published from pivotal phase III trials ADAURA, FLAURA, AURA 3 [54–57]. With a cut-off of 65 years, a significant benefit in terms of PFS was observed for osimertinib in all studies. Safety data based on age was not reported in these studies.

Several retrospective studies in a RWD have reported efficacy and safety data in older adult patients with osimertinib in untreated and pre-treated *EGFR*-mutated advanced NSCLC. Efficacy of osimertinib was in line with that observed in the pivotal trials, although the incidence of AEs seemed to be higher in older adult patients. HOT2002 is a first-line retrospective Japanese study in 132 patients ≥ 75 years with *EGFR*-TKI sensitizing mutations [58]. The median PFS was 19.4 months, with a better PFS in patients under 80 years of age. Grade ≥ 3 AEs were higher than in the FLAURA trial (42% *vs.* 34%). The most common AE was paronychia (44%), rash (39%), dry skin (39%) and anaemia (39%). The frequency of pneumonitis was 17% (grade ≥ 3 was 9%) with a mortality of 13%. A total of 41% required ≥ 1 dose reduction and 27% discontinued osimertinib, with pneumonitis the most common reason for discontinuation.

In pre-treated *EGFR* T790M mutation NSCLC patients, the ASTRIS trial reported similar outcomes in patients ≥ 75 years compared with < 75 years. Median PFS was 11.8 months (10.2–12.5) and 10.9 months (10.4–11.1) and estimated 1-year OS rate was 77% (68–79%) and 76% (74–78%), respectively [59]. Safety outcomes in the older adult population were not reported. In a phase II trial in 36 Japanese older adult patients (≥ 75 years) with T790M *EGFR* mutation, the median PFS was 11.9 months (95% confidence interval [CI] 7.9–17.5), and the median OS was 22.0 months (95% CI, 16.0-not reached [NR]) [60]. Most frequent AEs were fatigue (39%), anorexia (39%), diarrhoea (36%), rash (33%), paronychia (33%), haematological toxicity (40–48%) and hepatic toxicity (37–42%). There were no deaths, dose reductions nor discontinuations. Another study of Japanese patients included 17 patients ≥ 75 years, with no significant differences in terms of efficacy in these patients. Median PFS was 10.5 months (95% CI = 7.8–15.8) in the non-older adults’ group and 17.7 months (95% CI 8.4-NR, *p* = 0.11) in the older adults’ group, and median OS was NR (95% CI 15.5-NR) and 38.6 months (95% CI 14.3–52.8, *p* = 0.20), respectively. There were no differences in the frequency of AEs, except for grade ≥ 2 paronychia (2% vs. 17%, *p* = 0.04), and no differences in the incidence of dose reduction or discontinuation were observed [61]. GFPC 01-2016 included 43 octogenarian pre-treated patients that received osimertinib. Median PFS was 17.5 months (95% CI 12.2–19.0) and median OS was 22.8 months (95% CI 15.7–NR) [62]. Safety data was not reported.

#### Lazertinib

Lazertinib has been evaluated in a phase I/II trial in 78 pre-treated Asian patients with NSCLC harbouring the T790M *EGFR* mutation. However, efficacy and safety data based on the population age was not reported in this study [63].

### Atypical mutations

Atypical *EGFR* mutations comprise 30% of *EGFR*-mutated NSCLC and this is a heterogenous group with different sensitivities to *EGFR* TKI [64].

*EGFR* exon20 insertions (*EGFR*ex20ins) accounts for up to 12% of all *EGFR* mutations subtypes, representing the third most common *EGFR* mutation. Epidemiological characteristics of patients with *EGFR*ex20ins mirror those with common *EGFR* mutations. However, some cohorts have reported that patients with *EGFR*ex20ins NSCLC are older than patients with common *EGFR* mutations (67 years vs. 63 years, *p* = 0.01). NSCLC with *EGFR*ex20ins have a poor prognosis [65], due to limited activity of the approved *EGFR* TKIs [66], with only some clinical activity with osimertinib at double dose and no data about the efficacy or toxicity in *EGFR*ex20ins NSCLC according to age, because the sample size is too small [67]. Both the Food and Drug Administration (FDA) and the European Medicines Agency (EMA) have approved amivantamab in patients with platinum-refractory *EGFR*ex20ins NSCLC [68], whereas mobocertinib has been approved only by the FDA in the same setting [69]. These drugs have shown clinical activity in terms of RR (28–40%), median PFS (7.3–8.3 months) and OS (~ 2 years). Although both drugs had higher RR in younger patients, the cut-off was 65 years-old and median age was 62 years (42–84) in amivantamab and 60 years (27–84) in mobocertinib trial, respectively. Safety profile data according to age has not been reported [68, 69].

Uncommon *EGFR* mutations (G719X, S7681, L861Q and combinations) can occur more frequently in older adult patients (67.5 years) compared with other *EGFR* mutations [70]. However, this is not confirmed by other authors, who reported a median age of 60 years [71]. In current guidelines, afatinib and/or osimertinib are recommended for treatment of patients with these mutations [70–72], also because less data is available from other EGFR-TKIs. Unfortunately, no data about efficacy and safety is reported according to age.

### Combinations with antiangiogenic drugs and chemotherapy

Although initially effective, resistance to *EGFR* TKIs emerges within 10–12 months. There is also a proportion of patients with primary resistance [73–75]. Thus, to improve patient outcomes, new therapeutic strategies have been studied. Among them are *EGFR* TKIs combinations with both vascular endothelial growth factor (*VEGF*) inhibitors and chemotherapy.

In treatment-naïve patients, *EGFR* TKIs with platinum-doublet based chemotherapy compared with first-generation *EGFR* TKIs demonstrated improved OS, PFS and RR with the combination, though with grade ≥ 3 AEs [76–78]. The recent OPAL trial has addressed the combination of osimertinib plus platinum-pemetrexed chemotherapy in first-line. Although the results are encouraging (RR 91% and median PFS 31 months), 89.6% of patients experienced grade 3 AEs. Efficacy and toxicity regarding the age is not reported. Although efficacy results are encouraging, toxicity of combination strategy and the lack of direct comparison with osimertinib prevent their use as standard first-line therapy until the results of the phase III FLAURA2 trial (NCT04035486). Osimertinib plus bevacizumab was detrimental in second line compared to osimertinib alone [77]. The median age of the included population was < 65 years [76, 78]. There are no reports on safety data according to age. All these facts limit the applicability of these data to our population.

EGFR TKI combinations with both bevacizumab and ramucirumab have been studied. All RCT trials (except for one phase II) have shown improvement of PFS when compared with first-generation EGFR TKI as monotherapy, but they have not demonstrated an OS benefit [79–81]. Additionally, anti-VEGF drugs doubled high-grade toxicities. Efficacy data cut-off and median population age (65 years [57–71]) of the erlotinib-ramucirumab combination limit the evidence for this combination in our population [82]. In the RELAY study, the incidence of grade ≥ 3 AEs was higher in patients aged ≥ 70 than < 70 years in the ramucirumab plus erlotinib arm [83].

Diagnostic recommendations, management, and treatment.

Clinical guidelines strongly recommend performing a GA in all older adult patients with NSCLC, regardless of the genomic profile [84]. The GA should also therefore be considered in patients harbouring an *EGFR* mutation despite being candidates for *EGFR* TKIs, which have fewer toxic effects than platinum-based chemotherapy.

*EGFR* and other actionable genomic alterations should be tested in all patients diagnosed with non-squamous NSCLC, independently of the age at diagnosis [85]. Currently, multigene next-generation sequencing (NGS) is recommended in non-squamous NSCLC whenever possible [86]. In newly diagnosed patients with small tumour biopsies or lack of adequate tumour tissue for molecular testing, a liquid biopsy should be considered to assess *EGFR* status, either by single gene testing or by NGS [87]. Older adult patients with *EGFR* mutated MNSCLC should receive upfront targeted therapy with an *EGFR* TKI. In a pooled analysis of clinical trials evaluating first and second-generation *EGFR* TKIs, the benefit in terms of PFS was consistent in older adult patients (HR = 0.39, 95% CI 0.20–0.78) and in their younger counterparts (HR = 0.48, 95% CI 0.24–0.96) [88]. An exploratory subgroup analysis of the FLAURA study showed that frontline osimertinib yielded significant benefit in terms of PFS independently of the age at diagnosis [56]. Osimertinib has been considered the preferred treatment option in older adult patients with common *EGFR* mutations due to its favourable balance between efficacy and safety. Although safety data was not reported according to patients’ age in most trials, it is known that lung and cardiac toxicities associated with *EGFR* TKIs should be particularly monitored in this vulnerable population.

In patients with uncommon mutations such as G719X, S768I or L861Q, osimertinib and afatinib are the preferred treatment options, while in patients with an EGFRex20ins, amivantamab and mobocertinib have demonstrated treatment efficacy. However, data on efficacy and safety of those treatment strategies in the older adult population is still missing. Academic and collaborative initiatives to collect efficacy and safety data of *EGFR* TKIs in older adult patients with *EGFR* mutated NSCLC are therefore warranted and should be promoted by cooperative groups.

## Conclusions

The population is ageing. The incidence of certain diseases, such as cancer and particularly LC, increases with age. We consider that 70 years should be the threshold for including individuals in the older adult population; however, the cut-off used in most studies is 65 years. This limits the interpretation of the current available evidence. Older adult population with cancer requires a multidisciplinary approach with specific tools developed for geriatric medicine.

GA is beneficial during the LC diagnosis and treatment process, but most of the scientific evidence applies to patients who are candidates for chemotherapy. Data on the value of GA for patients with actionable genomic alterations, specifically *EGFR* mutations, are limited.

Given the efficacy of EGFR TKI in the older adult population and its favourable toxicity profile, it is essential to carry out mutational studies for these patients with LC, regardless of age and stage of LC. Applying GA in the population with *EGFR* mutation continues to be a healthcare challenge, given its lower level of scientific evidence and constraints in clinical practice. Prospective data on the value of GA in this population are also needed.

## Data Availability

Not applicable.
